# The role of chitin-rich skeletal organic matrix on the crystallization of calcium carbonate in the crustose coralline alga *Leptophytum foecundum*

**DOI:** 10.1038/s41598-019-47785-2

**Published:** 2019-08-15

**Authors:** M. Azizur Rahman, Jochen Halfar, Walter H. Adey, Merinda Nash, Carlos Paulo, Maria Dittrich

**Affiliations:** 1 Department of Chemical & Physical Sciences, University of Toronto at Mississauga, Toronto, Canada; 2 Department of Botany, Smithsonian Institution, Washington, DC 20560 USA; 3 Research School of Earth Sciences, Australian National University, Canberra, Australia; 4 Department of Physical and Environmental Sciences, University of Toronto Scarborough, Toronto, Canada

**Keywords:** Marine biology, Biodiversity

## Abstract

The organic matrix (OM) contained in marine calcifiers has a key role in the regulation of crystal deposition, such as crystalline structure, initiation of mineralization, inhibition, and biological/environmental control. However, the functional properties of the chitin-rich skeletal organic matrix on the biological aspect of crystallization in crustose coralline algae have not yet been investigated. Hence, the characterization of organic matrices in the biomineralization process of this species was studied to understand the functions of these key components for structural formation and mineralization of calcium carbonate crystals. We purified skeletal organic matrix proteins from this species and explored how these components are involved in the mineralization of calcium carbonate crystals and environmental control. Intriguingly, the analytical investigation of the skeletal OM revealed the presence of chitin in the crustose coralline alga *Leptophytum foecundum*. The sodium dodecyl sulfate-polyacrylamide gel electrophoresis (SDS-PAGE) analysis of the OM revealed a high molecular mass protein as 300-kDa. Analysis of glycosylation activity exposed two strong glycoproteins as 300-kDa and 240-kDa. Our study of the biominerals of live collected specimens found that in addition to Mg-calcite up to 30% aragonite were present in the skeleton. Our experiment demonstrated that the chitin-rich skeletal OM of coralline algae plays a key role in the biocalcification process by enabling the formation of Mg-calcite. In addition, this OM did not inhibit the formation of aragonite suggesting there is an as yet unidentified process in the living coralline that prevents the formation of aragonite in the living skeletal cell walls.

## Introduction

While abundant data is available on the organic matrices of corals, calcareous sponges, and molluscans^1–7^, very little is known about coralline algal organic matrices and their role in calcification. This is despite the fact that coralline algae are important ecosystem engineers with a high spatial coverage of both hard and soft substrate shallow benthic habitats in coastal areas worldwide^8^. Particularly, in the high latitudes of the Northern Hemisphere coralline algae are likely the single most common shallow benthic calcifier^9^. The organic matrix components contained in calcifying marine organisms play a key role in controlling the formation of calcium carbonate (CaCO_3_) minerals in the biomineralization process, particularly in response to changes of seawater chemistry^1,2,7,10,11^. When carbon dioxide (CO_2_) is absorbed by seawater, chemical reactions occur that reduce seawater pH, carbonate ion concentration, and saturation states of biologically important calcium carbonate minerals. Recent studies of the chemistry of global oceans have revealed that the changes in seawater chemistry over the course of geologic time have had a significant influence on the biomineralization of many marine organisms^2,12,13^.

The bulk of CaCO_3_ precipitation in seawater is skeletal and the crystalline polymorphs are calcite, vaterite, and aragonite. The most abundant calcium carbonate polymorphs in seawater reactions are aragonite and calcite^2,14–20^, and they are naturally distributed within the specific groups. We previously found chitin in the organic matrix of skeletons from a different type of coralline alga, *Clathromorphum compactum*, and the chitin appears to contribute to the biomineralization process^21^.

Chitin is one of the key components of cell walls and has been identified in bacteria, fungi, plants^22^. Ehrlich *et al*.^23^ reported the chitin for the first time in marine sponges and subsequently this biopolymer was recognized as an integral part in the calcification and silicification process of sponges^24^. The latter study also demonstrated the functional roles of chitin in relation to the templating properties in both calcification and silicification^25–30^. The functional and structural properties of chitin in coralline algae exposed similar features as the calcification process of sponges revealed.

Chitin also plays a vital role in the biomineralization system of diatoms^31^. Interestingly, both coralline and diatom have cell walls with a slight difference in the organic matrix components. Brunner *et al*.^32^ extracted chitin-containing biosilicates from the diatom *Thalassiosira pseudonana*. This study showed that cell walls in diatoms contain an organic network, which comprises crosslinked chitin fibers with biocilicates and other related organic components. However, organic components in the cell walls of coralline algae are different as they are mainly occupied by CaCO_3_ minerals^21^, specifically Mg-calcite, which, as shown below, could be formed by the influence of chitin-containing organic matrix.

The skeletal formation of biominerals is controlled by two interacting mechanisms: (1) the secretion of OM proteins and (2) the transportation of ions (e.g., magnesium, calcium, bicarbonates, and protons) to the site of mineralization (in the case of calcification)^2,33^. Organic matrix macromolecules, such as proteins and polysaccharides, play an important role in the process of biologically controlled calcification^2,34^. The OM is thought to regulate different facets of crystal deposition, such as the initiation of mineralization, assembly in crystalline structures and inhibition^2,34^. Thus, the skeletal OM plays a key role in the whole biomineralization process. In the present study, we investigated the role of skeletal organic matrix in biomineralization of the coralline alga *L*. *foecundum*. *Leptophytum foecundum* is a widely distributed Arctic species that extends into the subarctic photic zone^35^ and grows on pebbles and stones^36^. Specimens used in this study were live collected at 16–18 m water depth at Port Manvers Bay, Labrador, Canada (56°57.10 N, 61°32.80 W) in 2013.

Specifically, this study had two main goals. Firstly, to identify the composition of the organic matrix of *L*. *foecundum* and secondly to determine if or how the mineral formation is influenced by the skeletal organic matrix (OM). We carried out this analysis by applying ATR-FTIR (Attenuated Total Reflection-Fourier Transform Infrared), AFM (Atomic Force Microscopy), DXRxi Raman, SEM (Scanning Electron Microscope), XRD (X-ray Diffraction), SDS-PAGE (Sodium Dodecyl Sufate Polyacrylamide Gel Electrophoresis), and *In Vitro* crystallization techniques (see supplementary information for in-depth description of techniques). We then carried out an experiment adding the separated OM to aragonite and calcite solutions to determine the influence of OM on the mineral formation.

## Results

**Figure 1:**
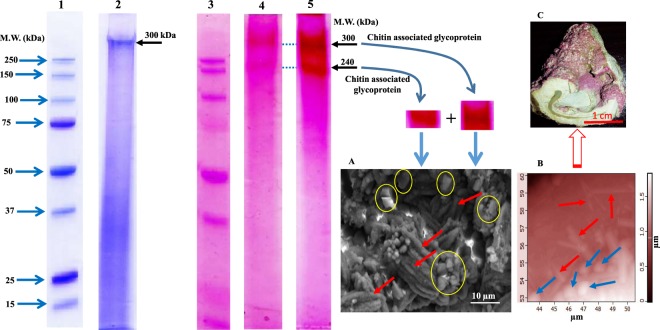
Biological function of OM in the formation of minerals. Electrophoretic analysis of skeletal matrix proteins extracted from coralline alga *L*. *foecundum*. Lanes 1 and 2 indicate the SDS-PAGE analysis with Coomassie Brilliant Blue (CBB) staining (see Fig. S3 for full-length gel image). Lane 1 shows the protein marker, Lane 2 shows purified skeletal matrix protein as 300 kDa. Arrows indicate protein bands. Lanes 3, 4 and 5 indicate the SDS-PAGE gel with Periodic Acid-Schiff (PAS) staining (see Fig. S4 for full-length gel images). Lane 3 shows the protein marker in PAS staining. Lanes 4 and 5 demonstrate very strong glycosylation activities with two highly abundant chitin associated glycoproteins (300 kDa and 240 kDa) in algal skeletons after purification of matrix protein. SEM image A, precipitation of minerals after introducing the chitin associated glycosylation OM components in the experimental design of skeletal formation. The red arrows and yellow circles indicate aragonite and calcite minerals, respectively. Image B, AFM of skeletal minerals with both calcite (blue arrows) and needle-like aragonite (red arrows) crystals on the surface (arrows). A finely polished skeletal sample was mapped using an AFM. Image C shows an original skeletal structure in the species *L*. *foecundum*, which was most likely formed by the major components of chitin associated glycoproteins containing in the OM.

**Figure 2:**
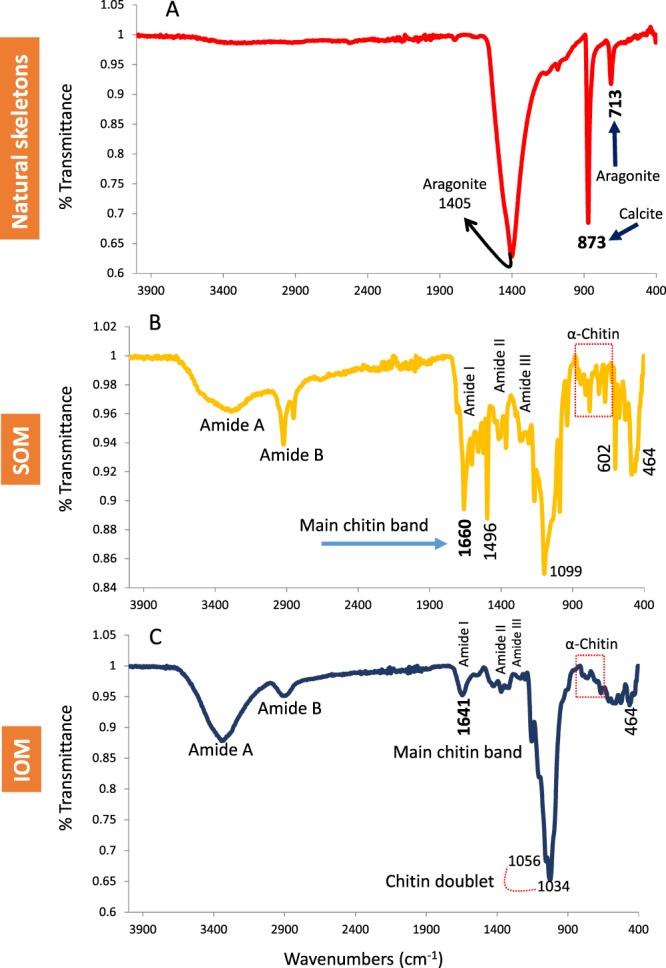
FTIR spectra of skeletons and OM. (**A**) FTIR spectra of powder samples of skeletons show calcite and aragonite bands (**B**,**C**) ATR-FTIR spectra of SOM and IOM extracted from skeletons of the coralline alga *L*. *foecundum* showed chitin bands.

Using SDS-PAGE analysis, we identified two high molecular masses of proteins as 300-kDa and 240-kDa (Fig. 1). Interestingly, both proteins appeared as glycoproteins, while 240-kDa did not appear in the Coomassie Brilliant Blue (CBB) staining, which means *L*. *foecundum* has a strong glycosylation activity. It is reported that the glycoprotein plays a key role to make the skeleton stronger^37^. We also confirmed that both these skeletal proteins are chitin associated (see Fig. 2 for details).

An ATR-FTIR analysis was performed to understand the structural properties of the OM components of the skeletal materials. Figure 2 shows the FTIR spectra of the soluble and insoluble organic matrices. The spectrum (Fig. 2A) reveals both calcitic and aragonitic skeletal components. In the analysis of lyophilized soluble organic matrix (SOM) (Fig. 2B) and insoluble organic matrix (IOM) (Fig. 2C), the IR showed structural protein bands as amide A (3000–3600 cm^−1^), amide B (2800–2950 cm^−1^), amide I (1550–1700 cm^−1^), amide II (1350–1590 cm^−1^) and amide III (1200–1250 cm^−1^), while the amide III is weaker in IOM than SOM. The main chitin band in SOM and IOM is present at 1660 cm^−1^ and 1641 cm^−1^, respectively. A strong band accredited to the chitin doublet (1056 and 1034 cm^−1^) is visible in the IOM in the range 1030–1110 cm^−1^ (stretching the C = O) (Fig. 2C). Another very strong band in SOM at 1099 cm^–1^ in the range 1030–1110 cm^−1^ (stretching the C = O) is also indicative of chitin (Fig. 2B). The IR spectra of both SOM and IOM in the fingerprint region (dashed red box in Fig. 2B, C) showed α-chitin formation^21,38^.

**Figure 3:**
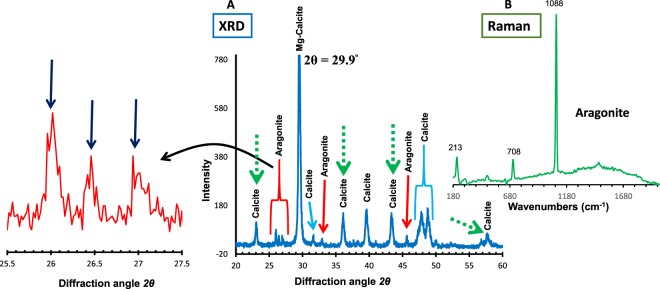
XRD pattern and Raman analysis of skeletons. (**A**) The XRD pattern of *2θ* scan identifies aragonite and Mg-calcite crystals formation in original skeletons. Green dashed arrows show chitin bands in the calcite planes. (**B**) Raman analysis of *L*. *foecundum* indicates aragonite minerals in skeletons. Raman analysis was conducted throughout skeletons with many specific spots.

**Figure 4:**
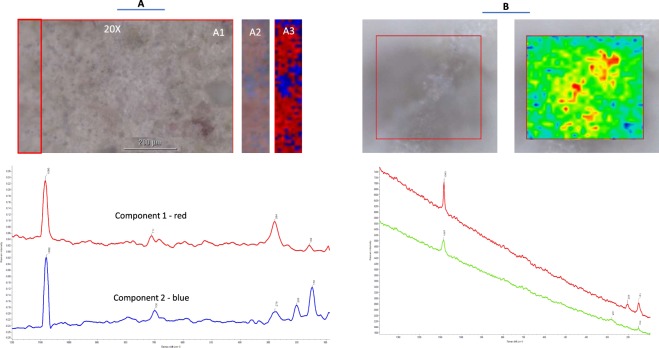
Raman spectroscopic imaging. **(A)** Raman Image and Multivariate Curve Resolution (MCR) analysis of powder sample. Light (A1) and Multivariate Curve Resolution (MCR) Raman (A2, A3) images of sample. The 2 components of MCR analysis corresponding to red and blue areas in image and showing, respectively, Raman peaks of Calcite (1086, 711, 284 and 158 cm^−1^) and Aragonite (1082, 700, 279, 205 and 150 cm^-1^). Blue areas corresponding to aragonite occupy about 29% of the total area of sample. **(B)** Detection of minerals in the skeleton. Representative averaged Raman spectra from red (aragonites) and green (calcites) areas of the image, respectively, showing a peak at 206 cm^-1^ and shift of most intense peaks from 1087 to 1083 cm^-1^. This provides evidence for the presence of aragonite in red areas. Spectra are shown on a common scale.

We performed an X-ray diffraction (XRD) analysis using the powdered samples of *L*. *foecundum* skeletal material to verify the purity of the mineral phases. The XRD indicated that the majority of the skeleton is Mg-calcite (Fig. 3A); however, a lesser amount (~29%) of crystal phases are aragonite (Fig. 3A,B). We randomly analyzed different portions of the skeletons and found aragonite scattered throughout. These results were supported by the confocal Raman spectroscopy of both powder and original skeletons (Fig. 4). In addition, atomic force microscope (AFM) images of the surface of the crust revealed the needle-like crystals typical of aragonite minerals (Fig. S1A,B). The characterization of organic matrix components revealed chitin bands in the calcite planes. Figure 3A shows the XRD pattern of both α and β-chitins (α-chitins in calcite planes 012, 110, 202; β-chitin in calcite plane 122)^21,38,39^

**Figure 5:**
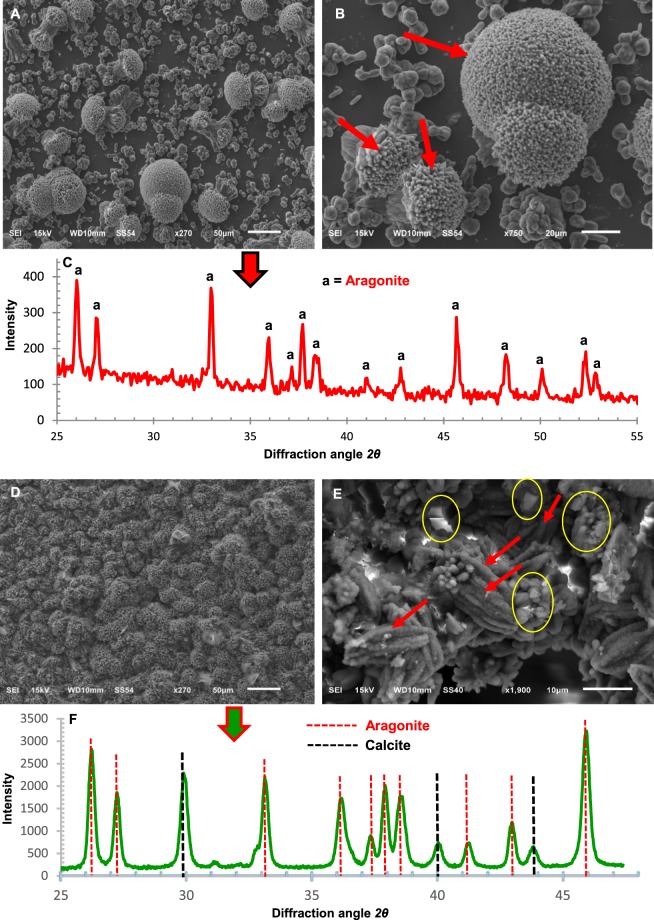
SEM images of aragonite and calcite grown *in vitro* and identification of polymorphism using XRD. (**A**) Crystals grown in aragonitic solution without any presence of OM; only aragonite crystals were grown. (**B**) An extended view of aragonite crystals, arrows indicate aggregation of aragonite. (**C**) Verification of aragonite crystals by XRD. (**D**) Crystals grown in aragonitic solution in presence of OM (containing 1.4 μg/mL proteins), 70% aragonitic and 30% calcitic crystals were formed. (**E**) An extended view of crystals. The red arrows and yellow circles indicate aragonite and calcite minerals, respectively. (**F**) XRD verification of experimental crystals formed demonstrating 70% aragonite and 30% calcite minerals (including the main band of Mg-calcite).

To enhance our understanding of how OM might influence biomineralization, we tested the influence of the OM on mineral formation in an aragonitic solution (50 mM MgCl_2_, Mg: Ca = 5:1) representing the modern seawater composition. In the control solution, only aragonite formed (Fig. 5A–C), however the aragonitic solution with OM (containing 1.4 µg/mL proteins) formed Mg-calcite (30%) as well as aragonite (70%) (Fig. 5D–F). Figure 5F shows the X-ray diffraction patterns of crystals in the skeletons. The diffraction angle, 2θ = 29.9° in the calcite (104) indicates Mg-calcite because the diffraction angle for pure calcite is 2θ = 29.3°^20,40^. These results indicate that OM can induce precipitation of Mg-calcite, but the presence of the OM does not inhibit the formation of aragonite. As per the ratio of magnesium in aragonitic solution, it was expected to form 100% aragonite, but due to the influence of OM up to 30% Mg-calcite was formed. The external morphology of the *L*. *foecundum* skeletons has been shown in Fig. S2. The detail analytical studies of this species in the skeletal, as well as in their decalcified state, might lead not only to suggestions about the structure of the organic skeletons but also to the way in which carbonate crystals are deposited.

## Discussion

In this study, we have identified the organic matrix and explored how functional organic molecules can regulate the biomineralization process of *L*. *foecundum*. We have confirmed the presence of chitin in *L*. *foecundum*. This is consistent with the finding of chitin in *Clathromorphum*^21^ and suggests that chitin is likely to be present in more genera of coralline algae. Although chitin is new in coralline algae, it has already been reported for other marine organisms as a universal template for biomineralization^24^. Chitin contained in the organic matrix serves as a template for preferential sites for nucleation and controlling the orientation and location of mineral phases^24^. It was also demonstrated that two minerals (amorphous and crystalline CaCO_3_,) were eventually formed from chitin^24^. The functional roles of chitin in the above are similar to our findings. In the present study, the experimental results showing that the OM enables the nucleation of Mg-calcite indicating that this OM likely plays a fundamental role in regulating the type of mineral formed by *L*. *foecundum* and specifically enabling Mg-calcite formation. Additional experimental work with other coralline genera is required before concluding that Mg-calcite formation is enabled in all coralline algae by the OM contained in their skeletons, however, our results suggest that is a distinct possibility. This is supported by experimental work by Raz *et al*.^41^ who demonstrated that organic extracts (not specifically identified) from articulated coralline algae enabled the increase in the amount of Mg incorporated into the Mg-calcite.

The presence of aragonite (approx. 29%) in the skeleton of *L*. *foecundum* raises a key question. How does this relatively high amount of aragonite nucleate and grow in the skeletons? Krayesky-Self, *et al*.^42^ reported in a different species that aragonite infills overgrown conceptacles and the amount of aragonite is a maximum 9.4% in one location and other locations are 0% to maximum 2.7%. They proposed “metabolites released by vegetative cells composing the conceptacle wall could influence the seawater chemistry and microenvironment within the conceptacles, favoring aragonite precipitation (Krayesky-Self, *et al*.)^42^”. In our study, the experimental results demonstrated that aragonite can be formed without the presence of OM (that is, in the control solution) and that the addition of the purified OM enabled nucleation of Mg-calcite but did not prevent the formation of aragonite. These findings are contrary to the proposal by Krayesky-Self *et al*., where they suggest a metabolic influence may favour aragonite precipitation.

From our results, it can be proposed that aragonite in *L*. *foecundum* can form where there is either no metabolic influence, such as in areas where a part of the crust is dead or in association with OM. Previous studies have shown the aragonite not only forms in emptied conceptacles but also in cell vacuoles of the crust near exposure to seawater such as at the base or in parts of the coralline crust that have been damaged by micro-borers^43^.This study was conducted with tropical coralline algae. However, there are no studies that have found aragonite forming as part of the coralline algal cell wall. This indicates that aragonite is completely inhibited from forming in these skeletal parts despite our experiment demonstrating aragonite can form in parallel with Mg-calcite. This inhibition suggests that perhaps it is only once the part of the crust is no longer metabolically active that aragonite can form, as is the condition in the experiment. Consequently, it can be surmised that there is an as yet unidentified process that completely inhibits aragonite formation in the actively forming skeletal parts.

Previously, Walker and Moss^44^ examined crustose coralline algal attachment related to the mineralization. They observed that upon the construction of space in between the attached crust and the substratum, aragonite crystals were precipitated below the crust and filled in the space helping in crust attachment. Walker and Moss^44^ proposed that metabolites secreted by the hypothelial cells may play a role in influencing aragonite precipitation. In this study, we did not specifically analyze OM from the hypothalial cells, so this proposal cannot be supported or refuted by our results. However, if the proposal by Walker and Moss is correct, that would suggest the metabolites secreted would have to differ from the main crust as otherwise Mg-calcite would be expected to form in parallel with the aragonite between the base and substratum.

Regarding the environmental control, Basso^45^ and Nelson^46^ have stated that coralline algae with their high magnesium calcite skeleton are more vulnerable to disbanding in response to increasing ocean acidification (OA) as compared to other marine calcifiers that precipitate aragonite. The findings in our study reveal that the relatively high amount of aragonite in the skeleton of *L*. *foecundum* may alter the dynamics of disbanding and may slow the dissolution response.

The control of mineral morphologies and structures in biological and synthetic systems has been discussed in detail^47^. It is well known that mollusks (e.g., Abalone shells) contain two distinct polymorphs (calcite and aragonite) of calcium carbonate. While *L*. *foecundum* also contains aragonite, there is no evidence that this aragonite forms as part of the living process as it does in mollusks, although it appears the formation of aragonite can occur in parallel with living processes continuing in other parts of the crust.

## Concluding Remarks

This new finding with the characterization of the skeletal organic matrix in another (previously reported^21^) coralline algal genus contributes substantially to our understanding of controls on coralline algal calcification. The key question now is whether chitin is common to all coralline algae. The experimental results also prompt the question of how aragonite formation might be inhibited from forming in the coralline cell wall and if this inhibition can break down under differing environmental conditions. The present results could influence our thinking about the future response of coralline algae to global climate change. Further functional studies of the effect of these organic matrix molecules (including chitin in coralline) on the nucleation and growth of calcite/aragonite crystals are required to build a deeper understanding of various biomineralization processes.

## Supplementary information

Supplementary info

## References

[1] Drake JL (2013). Proteomic analysis of skeletal organic matrix from the stony coral Stylophora pistillata. Proc Natl Acad Sci USA.

[2] Rahman MA, Oomori T, Worheide G (2011). Calcite formation in soft coral sclerites is determined by a single reactive extracellular protein. J Biol Chem.

[3] Miyamoto H (1996). A carbonic anhydrase from the nacreous layer in oyster pearls. Proc Natl Acad Sci USA.

[4] Aizenberg J, Ilan M, Weiner S, Addadi L (1996). Intracrystalline macromolecules are involved in formatethe morphogenesis of calcitic sponge spicules. Connect Tissue Res.

[5] Falini G, Weiner S, Addadi L (2003). Chitin-silk fibroin interactions: relevance to calcium carbonate formation in invertebrates. Calcif Tissue Int.

[6] Murray EJB, Murray SS, Simon R, Behnam K (2007). Recombinant Expression, Isolation, and Proteolysis of Extracellular Matrix-Secreted Phosphoprotein-24 kDa. Connect Tissue Res.

[7] Rahman MA (2014). Characterization of the proteinaceous skeletal organic matrix from the precious coral Corallium konojoi. Proteomics.

[8] Hofmann LC, Schoenrock K, de Beer D (2018). Arctic Coralline Algae Elevate Surface pH and Carbonate in the Dark. Front. Plant Sci..

[9] Adey WH, Halfar J, Williams B (2013). The coralline genus Clathromorphum foslie emend. adey: biological, physiological, and ecological factors controlling carbonate production in an arctic-subarctic climate archive. Smithsonian Contributions to the Marine Sciences.

[10] Falini G, Albeck S, Weiner S, Addadi L (1996). Control of aragonite or calcite polymorphism by mollusk shell macromolecules. Science.

[11] Falini G (2013). Control of aragonite deposition in colonial corals by intra-skeletal macromolecules. J Struct Biol.

[12] Stanley SM (2008). Effects of global seawater chemistry on biomineralization: past, present, and future. Chem Rev.

[13] Stolarski J, Meibom A, Przenioslo R, Mazur M (2007). A Cretaceous scleractinian coral with a calcitic skeleton. Science.

[14] Colfen H (2003). Precipitation of carbonates: recent progress in controlled production of complex shapes. Curr Opin Colloid In.

[15] Berner RA (1967). Thermodynamic Stability of Sedimentary Iron Sulfides. Am J Sci.

[16] De Leeuw NH (2002). Molecular dynamics simulations of the growth inhibiting effect of Fe^2+^, Mg^2+^, Cd^2+^, and Sr^2+^ on calcite crystal growth. J Phys Chem B.

[17] Stanley SM, Hardie LA (1998). Secular oscillations in the carbonate mineralogy of reef-building and sediment-producing organisms driven by tectonically forced shifts in seawater chemistry. Palaeogeogr Palaeocl.

[18] Kingsley RJ, Watabe N (1982). Ultrastructural Investigation of Spicule Formation in the Gorgonian Leptogorgia-Virgulata (Lamarck) (Coelenterata, Gorgonacea). Cell and Tissue Research.

[19] Rahman MA, Oomori T (2008). Structure, crystallization and mineral composition of sclerites in the alcyonarian coral. J Cryst Growth.

[20] Rahman MA, Oomori T (2009). *In Vitro* Regulation of CaCO_3_ Crystal Growth by the Highly Acidic Proteins of Calcitic Sclerites in Soft Coral, *Sinularia Polydactyla*. Connect Tissue Res.

[21] Rahman MA, Halfar J (2014). First evidence of chitin in calcified coralline algae: new insights into the calcification process of Clathromorphum compactum. Scientific reports.

[22] Banks IR (2005). A chitin synthase and its regulator protein are critical for chitosan production and growth of the fungal pathogen Cryptococcus neoformans. Eukaryot Cell.

[23] Ehrlich H (2007). First evidence of the presence of chitin in skeletons of marine sponges. Part II. Glass sponges (Hexactinellida: Porifera). J of Exp Zool. Part B, Mol and develop evol.

[24] Ehrlich H (2010). Chitin and collagen as universal and alternative templates in biomineralization. Int Geol Rev.

[25] Ehrlich H (2011). Calcite Reinforced Silica–Silica Joints in the Biocomposite Skeleton of Deep‐Sea Glass Sponges. Advanced Functional Materials.

[26] Klinger C (2019). Express Method for Isolation of Ready-to-Use 3D Chitin Scaffolds from Aplysina archeri (Aplysineidae: Verongiida) Demosponge. Marine Drugs.

[27] Shaala L (2019). New Source of 3D Chitin Scaffolds: The Red Sea Demosponge Pseudoceratina arabica (Pseudoceratinidae, Verongiida). Marine Drugs.

[28] Ehrlich H (2017). Isolation and identification of chitin from heavy mineralized skeleton of Suberea clavata (Verongida: Demospongiae: Porifera) marine demosponge. International Journal of Biological Macromolecules.

[29] Vacelet J, Erpenbeck D, Diaz C, Ehrlich H, Fromont J (2019). New family and genus for Dendrilla-like sponges with characters of Verongiida. Part I redescription of Dendrilla lacunosa Hentschel 1912, diagnosis of the new family Ernstillidae and Ernstilla n.g. Zoologischer Anzeiger v..

[30] Fromont J (2019). New family and genus of a Dendrilla-like sponge with characters of Verongiida. Part II. Discovery of chitin in the skeleton of Ernstilla lacunose. Zoologischer Anzeiger v..

[31] Ehrlich H., Witkowski A. (2015). Biomineralization in Diatoms: The Organic Templates. Biologically-Inspired Systems.

[32] Brunner E (2009). Chitin–based organic networks – an integral part of cell wall biosilica from the diatom *Thalassiosira pseudonana*. Angewandte Chemie International Edition.

[33] Allemand D (2004). Biomineralisation in reef-building corals: from molecular mechanisms to environmental control. Comptes Rendus Palevol.

[34] Weiner S, Hood L (1975). Soluble-Protein of Organic Matrix of Mollusk Shells - Potential Template for Shell Formation. Science.

[35] Adey W, McKibben D (1970). Studies on the maerl species *Phymatolithon calcareum* (Pallas) nov. comb. and *Lithothamnium coralloides* Crouan in the Ria de Vigo. Botanica Marina.

[36] Athanasiadis A, Adey WH (2006). The genus *Leptophytum* (Melobesioideae, Corallinales, Rhodophyta) on the Pacific coast of North America. Phycologia.

[37] Colfen H, Mann S (2003). Higher-order organization by mesoscale self-assembly and transformation of hybrid nanostructures. Angew Chem Int Edit.

[38] Cárdenas Galo, Cabrera Gustavo, Taboada Edelio, Miranda S. Patricia (2004). Chitin characterization by SEM, FTIR, XRD, and13C cross polarization/mass angle spinning NMR. Journal of Applied Polymer Science.

[39] Zhang Y, Xue C, Xue Y, Gao R, Zhang X (2005). Determination of the degree of deacetylation of chitin and chitosan by X-ray powder diffraction. Carbohydr Res.

[40] Rahman MA, Halfar J, Shinjo R (2013). X-Ray Diffraction Is a Promising Tool to Characterize Coral Skeletons. Advances in Materials Physics and Chemistry.

[41] Raz S, Weiner S, Addadi L (2000). Formation of High-Magnesian Calcites via an Amorphous Precursor Phase: Possible Biological Implications. Adv. Mater..

[42] Krayesky-Self S, Richards JL (2016). Aragonite infill in overgrown conceptacles of coralline *Lithothamnion spp*. (Hapalidiaceae, Hapaldiales, Rhodophyta): New insights in biomineralization and phylomineralogy. Journal of Phycology.

[43] Nash MC (2013). Dolomite-rich coralline algae in reefs resist dissolution in acidified condition. Nature climate change.

[44] Walker R, Moss B (1984). Mode of attachment of six epilithic crustose Corallinaceae (Rhodophyta). Phycologia.

[45] Basso D (2012). Carbonate production by calcareous red algae and global change. Geodiversitas.

[46] Nelson WA (2009). Calcified macroalgae, critical to coastal ecosystems and vulnerable to change: a review. Marine Freshwater Research.

[47] Meldrum FC, Colfen H (2008). Controlling mineral morphologies and structures in biological and synthetic systems. Chem Rev.

