# Supplementary material for: The role of chitin-rich skeletal organic matrix on the crystallization of calcium carbonate in the crustose coralline alga Leptophytum foecundum
Source: Sci Rep. 2019 Aug 15;9:11869. doi: 10.1038/s41598-019-47785-2 (PMC6695481; doi:10.1038/s41598-019-47785-2)

Supplementary information

The role of chitin-rich skeletal organic matrix on the crystallization of calcium carbonate in the crustose coralline alga *Leptophytum foecundum*

M. Azizur Rahman[1, 4*], Jochen Halfar[1], Walter H. Adey[2], Merinda Nash[3], Carlos Paulo[4], and Maria Dittrich[4]

[1]Department of Chemical & Physical Sciences, University of Toronto at Mississauga, Canada

[2]Department of Botany, Smithsonian Institution, Washington, DC, 20560, USA

[3]Research School of Earth Sciences, Australian National University, Canberra, Australia

[4]Department of Physical and Environmental Sciences, University of Toronto Scarborough, Canada

*Correspondence to M. Azizur Rahman (aziz@climatechangeresearch.ca or, mazizur.rahman@utoronto.ca)

Materials and methods

Experimental specimens

Samples of *Leptophytum foecundum* were collected on 22 July 2013, from 16 to 18m depth in inner Port Manvers Bay, Labrador, Canada (56◦57.10N, 61◦32.80W). In this area, sea ice is extensive from November through early July, although in inter-island, rapid current areas, small ice-free areas may occur even in mid-winter. Water temperature measured at the site on 22 July 2013 was 0.5◦C on the bottom, although this is relatively early in the warm season. At the collection site, the bottom consisted of extensive shell–pebble (gravel) bed largely encrusted with *Leptophytum laeve*, *Leptophytum foecundum* and *Clathromorphum compactum* with scattered rhodoliths of *Lithothamnion glaciale* and *Lithothamnion tophiforme*. Some of the *C. compactum* crusts were several centimeters in thickness and the *Leptophytum foecundum* ranged up to several mm in thickness. Nash and Adey 2017[1] describe the site in greater detail.

Leptophytum foecundum has been placed in the same genus as the widespread Arctic/Subarctic *Leptophytum leave*. However, as Adey et al 2015[2] have shown with DNA analysis, it belongs in a separate genus. At this time, that change has not been made. *L. foecundum* is known to occur from the western Canadian Arctic across the Arctic Ocean and adjacent North Atlantic to Novoya Zemlya in Russia. It extends the furthest south in the Gulf of Maine in the western North Atlantic (Athanasiadis and Adey, 2006)[3].

Typically, *L. foecundum* is a thin crust, approximately one-half hypothallium and one-half perithallium, with distinctive, raised and rimmed conceptacles. However, as shown by Athanasiadis and Adey 2006[3], *L. foecundum*, especially in the form of? rhodoliths and on pebble bottoms, tends to repeatedly overgrow itself, not only earlier-formed crusts but also the postmature conceptacles on those crusts. In so doing, it can form crusts that are one cm in thickness or greater. However, unlike the *Clathromorphum* crusts, which are typically formed by continuously growing perithallium, thick *L. foecundum* crusts are repeated hypothallium and perithallium units, sometimes with entrained foreign inclusions and spaces. Care must be taken to link anatomy with chemistry in defining bulk chemistry in this species.

Preparation of skeletons

To ensure contamination-free of samples of other associated tissues (e.g., fungus, bacteria etc.) or other skeletal minerals (e.g., shells, corals etc.), the preparation of skeletons of the algal body was performed very carefully. We used both upper and lower levels (lower level normally stays attach to rock or other organisms in the sea) to confirm the skeletal mineral. We used a series of mechanical and chemical treatments. (1) Experimental skeletons were made small pieces and all pieces were washed with 95% ethanol (five times) and distilled water (ten times) while stirring; (2) The cleaned skeletons were dried and examined using a microscope to determine whether they were completely free of unwanted tissues and other contaminants. We also confirmed via Scanning electron microscope (SEM) that all skeletons are completely contamination-free; (3) The selected skeletons were finely ground; (4) Skeletal powder was stirred with 1 M NaOH for 2 h; (5) To ensure again that the skeletons were tissue free (especially fungus and bacteria), they were stirred vigorously in a 10% sodium hypochlorite (NaOCl) bleaching solution for 1 h to remove fleshy tissues and debris. The treated samples were washed

with distilled water until all chemicals were removed. Finally, the samples were washed with MilliQ water five times. All steps in the preparation of skeletons were conducted at room temperature, and the materials obtained were stored at 4°C until further analysis.

Preparation of soluble and insoluble organic matrices

The mechanically and chemically cleaned alga (ca. 10 g) were decalcified in 0.5 M EDTA-4Na (pH 7.8) overnight. The decalcifying solution was centrifuged (Eppendorf-5430) at 4,000 rpm (15 minutes), and the soluble organic matrix (SOM) in the supernatant was filtered and collected for further purification. The insoluble organic matrix (IOM) in the precipitate was subsequently lyophilized and washed with distilled water (5 times). The solution was dialyzed prior to lyophilization to remove EDTA against 5x1 L of distilled water for 64h while water was changed five times using dialysis tubing. Additional details on sample preparation can be found in Rahman et al[4,5]. To purify SOM, filtrated samples were passed through two Sep-Pak C_{18} cartridges connected in tandem (Waters Associates, Milford, MA) to remove the EDTA completely and separate the soluble macromolecules, followed by passing through 10% acetonitrile (2 mL/three times). Finally, the absorbed macromolecules were eluted in 50% acetonitrile (2 mL/three times), frozen in a deep freezer and lyophilized. Deacetylation with NaOH (see preparation of skeletons above) and decalcification of the calcified skeletons following the above-mentioned procedures are perfect to obtain high content of soluble chitin from the skeletal samples of coralline algae.

Analysis of organic matrix proteins (OMP)

The Precision Plus SDS-PAGE standard (Bio-Rad) was used as a protein marker for electrophoresis analysis. An eluate (derived from 5 g of the algal skeleton) was run on 12% polyacrylamide gel with several replications. The purification of the organic matrix (OM) and total OMP were done following the methods of Rahman et al., (2011)[4]. Periodic acid-Schiff (PAS) staining was used to identify glycoproteins, and the two-high abundant chitin associated glycoproteins were identified to be the only OMPs occluded in the algal skeletons of *L. foecundum*.

Preparation of the solution for in vitro crystallization

To investigate the effect of matrix proteins on the $CaCO_3$ formation, *in vitro* crystallization experiments was carried out using a solution containing the components typically present in seawater. Two crystallization solutions were prepared, one that induces calcite (a calcitic crystallization solution) and one that induces aragonite (an aragonitic crystallization solution). The calcitic solution consisted of a supersaturated solution of Ca $(HCO_3)_2$ that was prepared by purging a stirred aqueous suspension of $CaCO_3$ with carbon dioxide. Calcium carbonate was dissolved in CO_2-aerated water, and excess precipitates were removed by filtration using filter paper (Whatman, 0.22 μm). The aragonitic solution (Mg: Ca = 5:1) was obtained by adding 50 mM $MgCl_2$ to the calcitic solution. Crystallization experiments were carried out in each solution with and without SOM extracted from the algal skeletons. The solutions were stirred for 10 min in a beaker and allowed to sit for 15 days without further manipulation.

Raman Spectroscopy and Microscopy

Two kinds of samples were analyzed using Raman spectroscopy, skeletal powder, and original skeletons. Prior to conduct Raman analysis, we ensured that both powder and skeletons were contamination free by other associated tissues or other skeletal minerals as described in the "Preparation of skeletons section" above. The samples were analyzed using Thermo Fisher Scientific DXRxi Raman Imaging Microscope configures with 455 nm or 532 nm laser and a full range grating providing 5 cm^{-1} spectral resolution. The samples were fixed on a standard microscope glass slide using double-sided adhesive tape. To analyze by Raman spectroscopy with the original skeletons, several small pieces with a smooth surface were selected from the middle part of the skeletons. A different type of Raman spectroscopy was also used to investigate the samples. In this case, all measurements were obtained in a NTEGRA Spectra system (NT-MDT, Zelenograd/Moscow, Russia) coupled with a Solar TII Spectrometer. This system is equipped with an upright confocal laser microscope, a photomultiplier tube (PMT) and a Raman spectrograph coupled with a high-resolution CCD camera (1600 x 200 pixels). PMT imaging was used to find the areas of interest for Raman spectra acquisition. Raman spectra were obtained at room temperature and at ambient pressure using a 532 nm wavelength laser with a maximal power of 8.7 mV. Backscattered light was collected through a 100x objective, 0.7

numerical aperture (NA) and redirected to the CCD camera (cooled at -70 ºC). The spectra were obtained with a spectral resolution of 1.6 cm^{-1} for a 600 lines/mm grating.

In Situ AFM

The finely polished samples were used for the Atomic Force Microscope (AFM) analysis. The topography of the surface was mapped using an AFM in phase imaging mode. Phase images can be generated as a consequence of variations in material properties such as friction. AFM observations were conducted with an Atomic Force Microscopy NT-MDT Ntegra Spectra at room temperature.

Infrared (IR) Spectrometry

Fourier transform infrared spectroscopy (FTIR) analyses were directly performed on finely powdered *L. foecundum* skeletal material. Powdered samples of skeletons and KBr (potassium bromide) were mixed (about 5% powdered samples in KBr) and loaded into the sample holder. A background spectrum was measured for KBr.

SOM (soluble organic matrix) and IOM (insoluble organic matrix) have different properties and were therefore prepared differently for analysis by Attenuated Total Reflection-Fourier Transform Infrared (ATR-FTIR) spectroscopy to understand the details of structural and functional properties of the skeletal material. Both SOM and IOM were subjected to ATR-FTIR using an FTIR spectrometer (Model Alpha, Bruker) equipped with a diamond ATR crystal cell (45˚ ZnSe; 80 mm long, 10 mm wide and 4 mm thick). For spectral analysis, the SOM and IOM lyophilized samples were placed onto the crystal cell and the cell was clamped into the mount of the FTIR spectrometer. Spectra in the range of 400–4000 cm^{-1} with automatic signal gain were collected in 32 scans at a resolution of 4 cm^{-1} and were rationed against a background spectrum recorded from the clean empty cell.

X-Ray Diffraction (XRD)

The same finely ground skeletal powder samples as for FTIR were used for this analysis. The polymorphism of crystals in the skeletons was determined by an X-ray diffractometer (Phillips) with 30 kV and 40 mA Cu Kα radiations. Sample analyses were carried out in the 20°–60° range of 2θ angle, with step sizes of 0.020° and a point measurement time of 2 s.

Precipitated crystals were collected from the bottom of a beaker, each experiment was performed separately. Prior to collect the samples from the bottom of the beaker, the crystals were lightly rinsed with distilled water to avoid any further reaction and contamination. The crystals were air-dried, finely ground using a hand grinder, and examined under a light microscope. The polymorphism of the crystals was then determined by a Siemens D5000 Bragg-Brentano Diffractometer.

Scanning Electron Microscopy (SEM)

Observation of Skeletal Structure: In order to understand the skeletal structure, *L. foecundum* samples were subjected to observation under SEM (JSM6610LV; JEOL operated at 15 kV, WD14 mm). Prior to that samples were treated to remove any contamination as mentioned above (see preparation of skeletons) and SEM observations were made prior to grinding. All specimens were placed in a sample holder and coated with a palladium-gold mixture.

Characterization of $CaCO_3$ Crystal Growth: Different concentrations of the skeletal organic matrix (OM) were added to the aforementioned calcite and aragonite solutions, and the resulting crystals were analyzed by SEM to determine the role of organic matrix in the biomineralization process of *L. foecundum*. After SOM addition and subsequent mixing (10 minutes with stirring), SEM glass plates (13 m/m) were placed on the bottom of the sample beakers to collect precipitated crystals. After completion of the *in vitro* experiments, the glass plates were then collected and lightly rinsed with distilled water to avoid any contamination and to stop the further reaction. All specimens were air-dried and coated with a palladium-gold mixture. The morphologies and different shapes of the crystals were then observed by SEM. The morphology of crystals formed in the presence of OM (affected crystals) was compared with that of crystals grown without OM (control crystals). The samples were examined using a scanning electron microscope (JSM6610LV; JEOL) operated at 15 kV.

Supplementary Figure legends

Fig. S1. Atomic force microscopy images of algal skeletons. (A) Needle-like aragonite crystals on the surface (arrows). (B) AFM of the 3D structure with needle-like aragonite crystals on the surface (arrows).

Fig. S2. Portions of skeleton of *L. foecundum*. (A) A skeletal shape in the direction of up shows structural design on the surface of the species. (B) A skeletal section which usually stays in the direction of down in attachment with other species or rocks in the sea. (C) Two portions of skeletal shape with the direction of up.

Fig. S3. Electrophoretic analysis of skeletal matrix proteins extracted from coralline alga *L. foecundum*. Lanes 1 and 2 indicate the SDS-PAGE analysis with Coomassie Brilliant Blue (CBB) staining. Lane 1 shows the protein marker, Lane 2 shows purified skeletal matrix protein as 300 kDa. Arrows indicate protein bands.

Fig. S4. SDS-PAGE gel with Periodic Acid-Schiff (PAS) staining. Lane 1 shows the protein marker in PAS staining. Lanes 2 and 3 demonstrate strong glycosylation activities with two highly abundant chitin associated glycoproteins (300 kDa and 240 kDa) in algal skeletons after purification of matrix protein. Lanes (out of dashed box) showed additional bands of glycoproteins (300 kDa and 240 kDa) and protein marker. Arrows indicate protein bands.

Supplementary References

1. Nash, Merinda C. and Adey, Walter H. Anatomical structure overrides temperature controls on magnesium uptake -- calcification in the Arctic/subarctic coralline algae *Leptophytum laeve* and *Kvaleya epilaeve* (Rhodophyta; Corallinales). Biogeosciences 15: 781-795 (2018).
2. Adey, W., J. J. Hernandez-Kantun, G. Johnson, and P. Gabrielson. DNA sequencing, anatomy and calcification patterns support a monophyletic, Subarctic, carbonate reef-forming *Clathromorphu*m (Hapalidiaceae, Corallinales, Rhodophyta). Journal of Phycology 51:189 (2015).
3. Athanasiadis, A. and W. Adey. The genus *Leptophytum* (Melobesioideae, Corallinales, Rhodophyta) on the northern Pacific Coast of North America. Phycologia 45:71-115 (2006).
4. Rahman, M. A., Oomori, T. & Worheide, G. Calcite formation in soft coral sclerites is determined by a single reactive extracellular protein. *J Biol Chem* 286, 31638-31649 (2011).
5. Rahman, M. A., Isa, Y. & Uehara, T. Proteins of calcified endoskeleton: II Partial amino acid sequences of endoskeletal proteins and the characterization of the proteinaceous

organic matrix of spicules from the alcyonarian, *Sinularia polydactyla*. *Proteomics* 5, 885-893 (2005).

Fig. S1

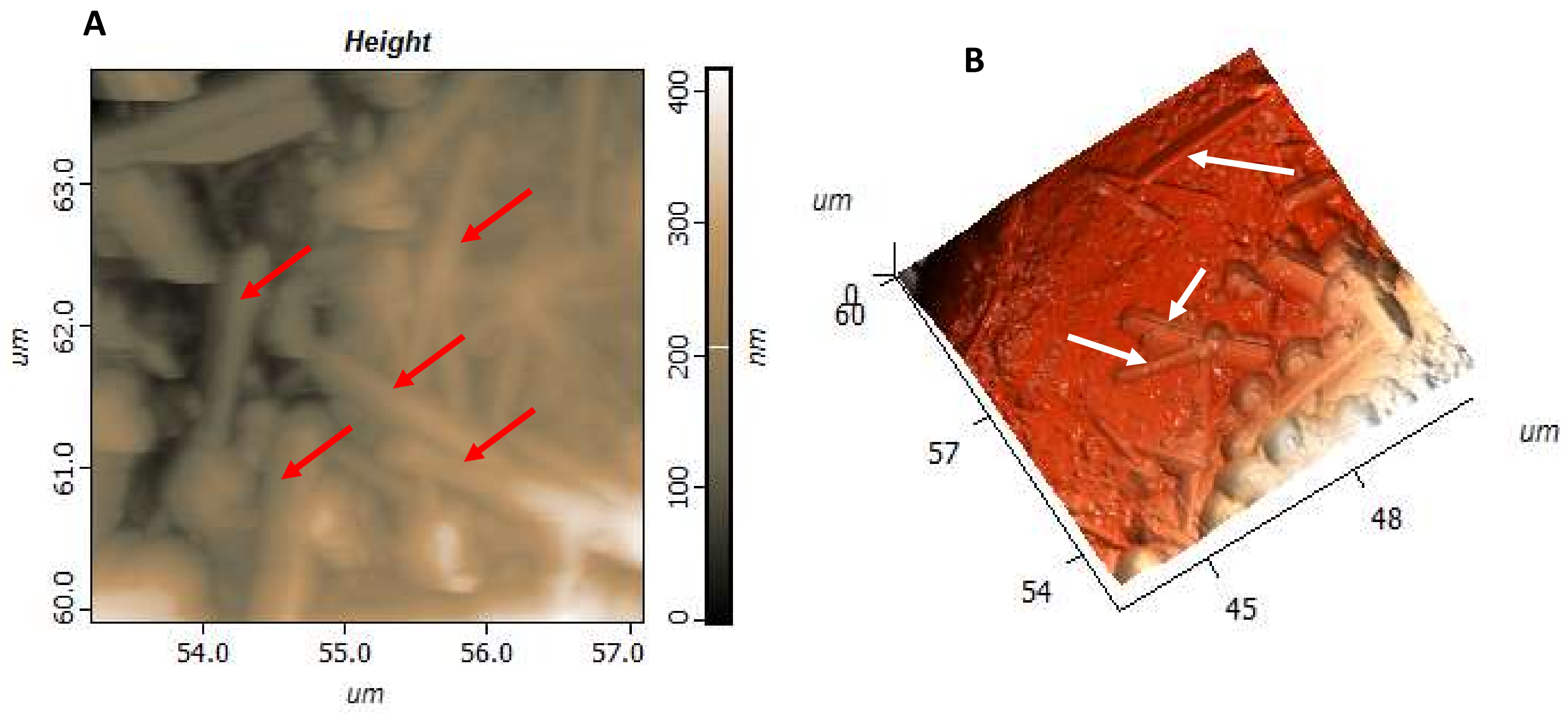
A
Height
63.0
62.0
61.0
60.0
um
54.0
55.0
56.0
57.0
um
400
300
200
100
0
nm
B
um
0
60
57
54
45
48
um

Fig. S2

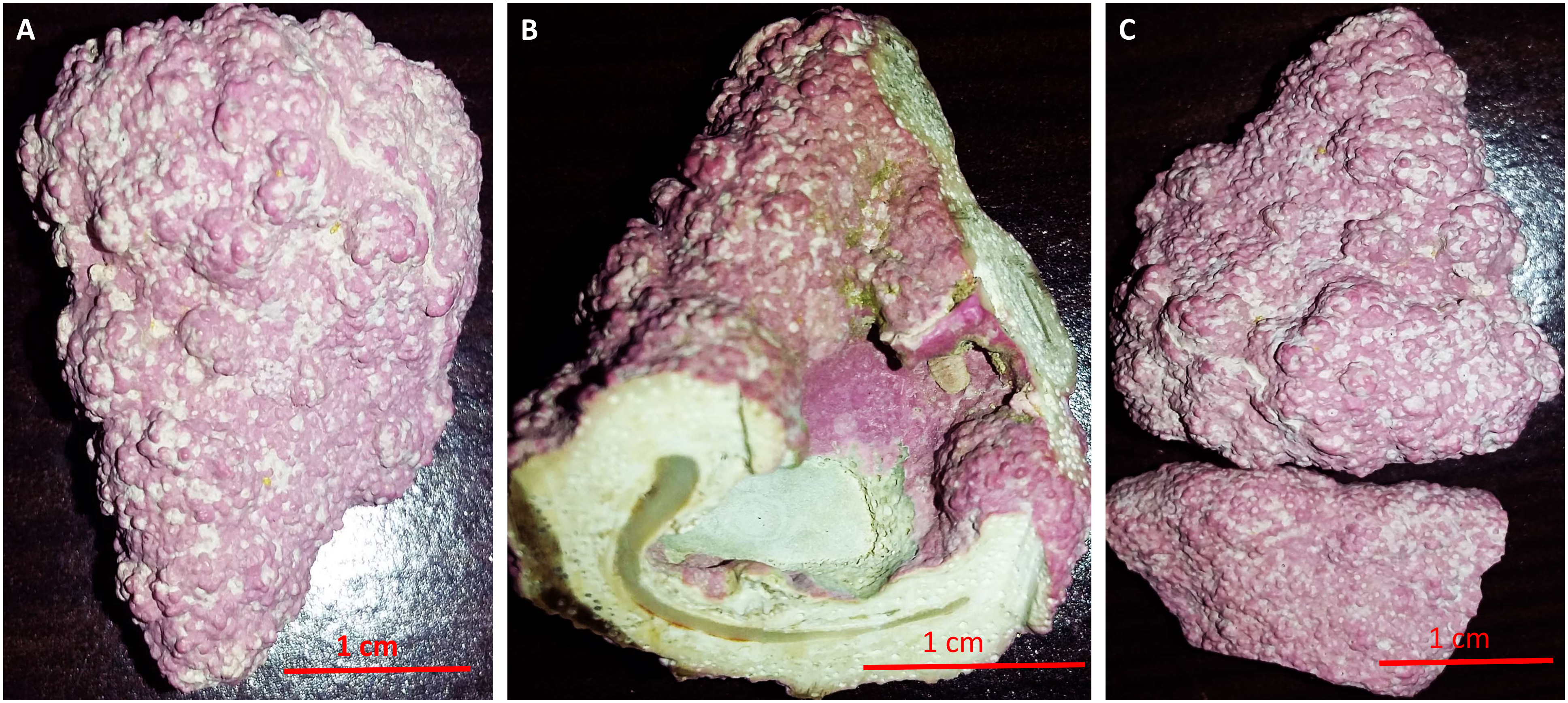

Fig. S3

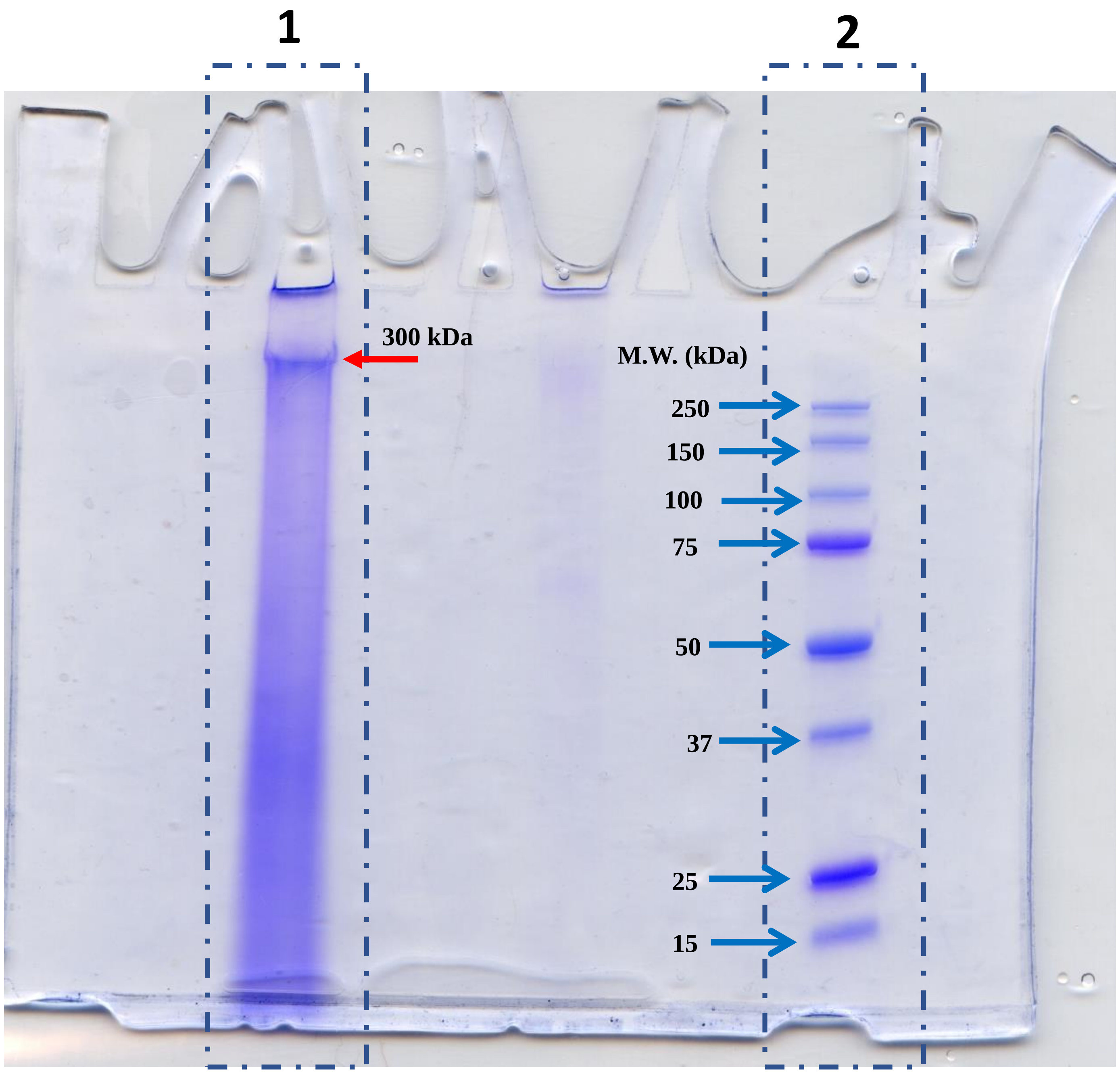

Fig. S4

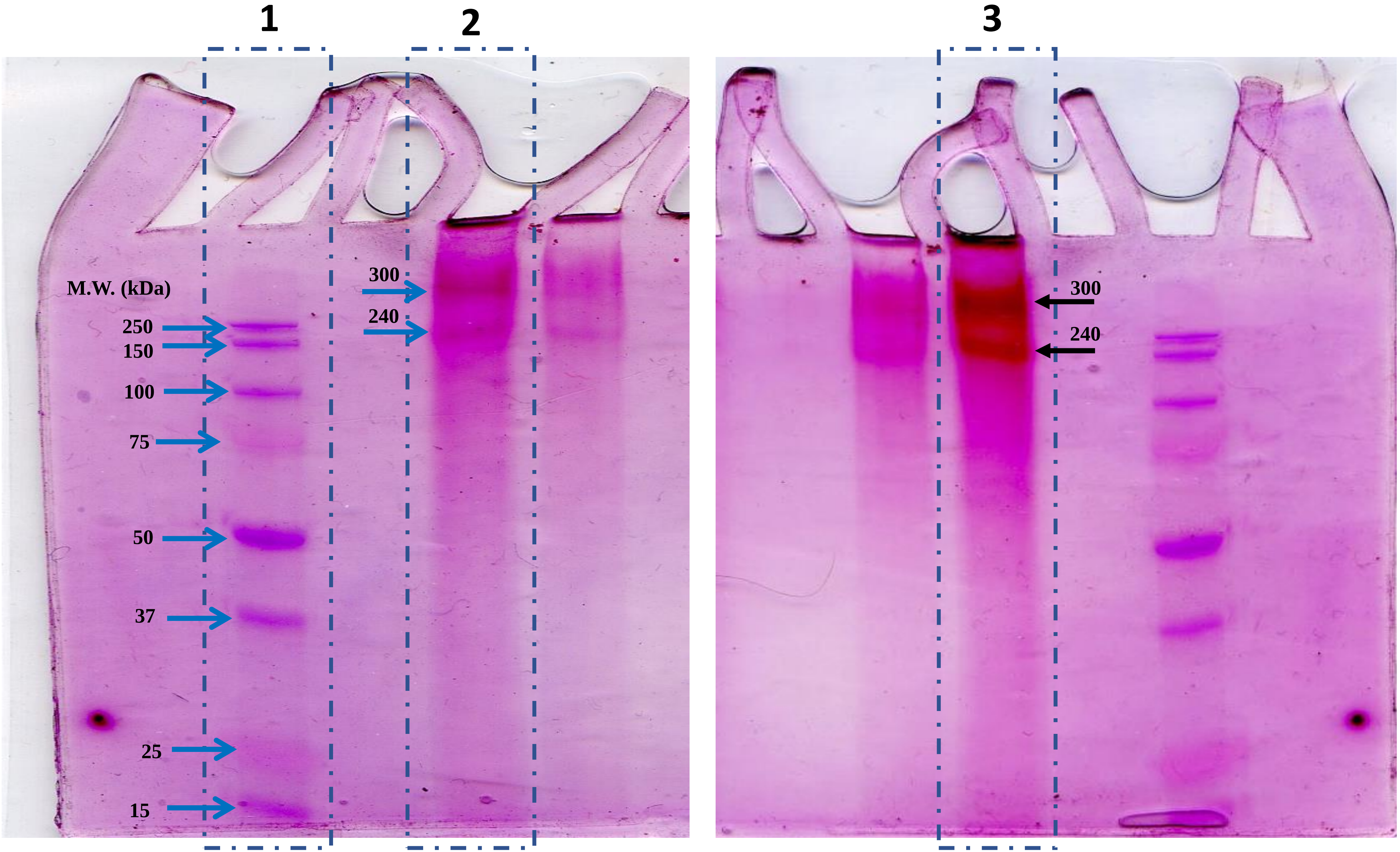

Supplement: Supplementary file 1 — Supplementary info [file 41598_2019_47785_MOESM1_ESM.pdf]
